# Streamlining Alzheimer’s disease diagnosis: real-world validation of two-cut-off diagnostic models based on plasma p-tau/Aβ42 ratios

**DOI:** 10.1007/s00415-026-13833-x

**Published:** 2026-05-08

**Authors:** Martina Poli, Chiara Giuseppina Bonomi, Martina Gaia Di Donna, Ilaria Barberis, Emanuele Luca Ginevra, Marzia Nuccetelli, Sergio Bernardini, Diego Centonze, Alessandro Martorana, Caterina Motta

**Affiliations:** 1 Memory Clinic and Neurodegenerative Dementia Research Unit, Policlinico Tor Vergata, University of Rome “Tor Vergata”, viale Oxford 81, 00133 Rome, Italy; 2 IRCCS Neuromed, via Atinense 18, 86077 Pozzilli, IS Italy; 3 Department of Clinical Biochemistry and Molecular Biology, Policlinico Tor Vergata, University of Rome “Tor Vergata”, viale Oxford 81, 00133 Rome, Italy; 4 Neurology Unit, Policlinico Tor Vergata, University of Rome “Tor Vergata”, viale Oxford 81, 00133 Rome, Italy

**Keywords:** Alzheimer’s disease, Blood-based biomarkers, Plasma phosphorylated tau, Plasma combined ratios, Real-world clinical implementation

## Abstract

**Introduction:**

Anti-amyloid monoclonal antibodies have increased the need for scalable, minimally invasive biomarkers for Alzheimer’s disease (AD). In this single-center cohort study, we evaluated plasma biomarkers performance in detecting biologically defined AD, assessing diagnostic accuracy and generalizability outside dedicated laboratory settings and exploring suitability for clinical implementation.

**Methods:**

We enrolled 204 outpatients referred to the memory clinic of Policlinico “Rome Tor Vergata” who underwent standard work-up, lumbar puncture for cerebrospinal fluid (CSF) biomarkers and paired blood sampling. Among plasma biomarkers, phosphorylated tau (p-tau) 181, p-tau217, and their ratios adjusted for Aβ42 were measured on the Lumipulse platform. AD pathology was defined by CSF p-tau181/Aβ42 ≥ 0.069. ROC analyses estimated AUCs, and a two-cut-off approach targeting 90% sensitivity and specificity classified individuals as low, intermediate, or high AD risk. Subgroup analyses examined the impact of sex, age (< 75, ≥ 75 years), chronic kidney disease, and cognitive impairment (MMSE ≥ 26/30, < 26/30) on plasma biomarker levels.

**Results:**

Among single analytes, plasma p-tau217 showed the highest discriminative capacity (AUC 0.883). Combined ratios improved overall performance (p-tau181/Aβ42, AUC 0.928; p-tau217/Aβ42, AUC 0.894) and reduced intermediate-risk classifications to < 15%, with slightly better performance in women, patients < 75 and cognitively unimpaired. The two-cut-off model improved accuracy and rule-in ability.

**Discussion:**

Plasma p-tau/Aβ42 ratios show high and robust accuracy for detecting CSF-defined AD pathology. A two-step approach based on p-tau181/Aβ42 or p-tau217/Aβ42 could streamline diagnostic workflows in memory clinics, reserving second-line assessments to indeterminate cases and supporting selection of candidates for disease-modifying anti-amyloid therapies.

**Supplementary Information:**

The online version contains supplementary material available at 10.1007/s00415-026-13833-x.

## Introduction

In recent decades, fluid and imaging biomarkers have transformed the diagnostic approach to Alzheimer’s disease (AD), which is now conceived as a clinico-biological continuum, beginning with neuropathological changes in asymptomatic individuals and progressing to overt cognitive symptoms [1]. Cerebrospinal fluid (CSF) biomarkers together with amyloid positron emission tomography (PET) remain the gold standard for the in vivo diagnosis of AD [2], with the revised criteria favoring hybrid ratios of CSF biomarkers (e.g., CSF Aβ42/40, p-tau181/Aβ42, t-tau/Aβ42) over stand-alone measures. Notably, the CSF p-tau181/Aβ42 ratio is highly informative, with robust ability to detect AD and superior performance compared with both the Aβ42/40 ratio and p-tau181 alone [3]. However, the invasiveness, costs, and limited availability of traditional biomarkers outside specialized care settings have prompted a shift toward more accessible biomarkers [4].

Blood-based biomarkers (BBBs) have gained growing attention for their feasibility and scalability, leading the Alzheimer’s Association Workgroup to include them in the most recent diagnostic criteria for AD [5]. Specifically, plasma p-tau assays have demonstrated robust clinical performance in both clinical trials and observational studies, and p-tau217 assays—alone or used in combined ratios—achieve a diagnostic accuracy comparable to that of approved CSF assays [6]. A two-step diagnostic workflow has been proposed in which plasma p-tau217 serves as a first-line test to detect AD-related biological changes in patients with cognitive symptoms [7]. This strategy should reduce the need for confirmatory CSF testing to uncertain cases and aligns with current recommendations for integrating plasma biomarkers into clinical practice [8]. Moreover, replacing p-tau217 alone with the p-tau217/Aβ42 ratio substantially has been found to decrease the proportion of patients falling into an “uncertain risk” category for Aβ positivity [9]. Following this evidence, the plasma p-tau217/Aβ42 ratio Lumipulse G platform-based assay has received Breakthrough Device Designation from the U.S. Food and Drug Administration, and has been submitted as a commercially available blood-based in vitro diagnostic test for AD.

While these findings are promising and consistent, most evidence still derives from experimental cohorts, and only a limited number of studies have evaluated the performance of plasma biomarkers in real-world populations [10–12]. Systematic data on reproducibility in routine analyses performed in local laboratories are lacking. Furthermore, although accurate plasma biomarkers show excellent performance when predefined cut-offs are used, their implementation in non-specialist settings is hampered by the absence of standardized validation procedures and by the potential impact of common medical comorbidities on test interpretation [13].

Against this background, the aim of the present study was to assess the performance of plasma biomarkers in a real-world memory clinic cohort for the biological diagnosis of AD, to evaluate the diagnostic accuracy and generalizability when these assays are performed routinely in non-dedicated laboratory settings, and to explore their suitability for implementation in clinical practice.

## Materials and methods

### Enrollment of patients

Between January 2024 and July 2025, we enrolled 325 consecutive patients referring to the UOSD Centro Demenze of the University Hospital “Policlinico Tor Vergata” in Rome. Due to suspected neurodegenerative disease, patients underwent a complete neuropsychological assessment [14], including Mini-Mental State Examination (MMSE), comprehensive laboratory testing, brain magnetic resonance imaging, and 18-Fluorodeoxyglucose PET.

Patients were considered eligible if they had a diagnosis of: (1) mild dementia due to AD (18 < MMSE ≤ 24) [15]; (2) Mild Cognitive Impairment (MCI) due to AD (MMSE > 24) [16]; (3) behavioral variant frontotemporal dementia (bvFTD); (4) primary progressive aphasia (PPA); (5) dementia with Lewy bodies (DLB).

**Fig. 1:**
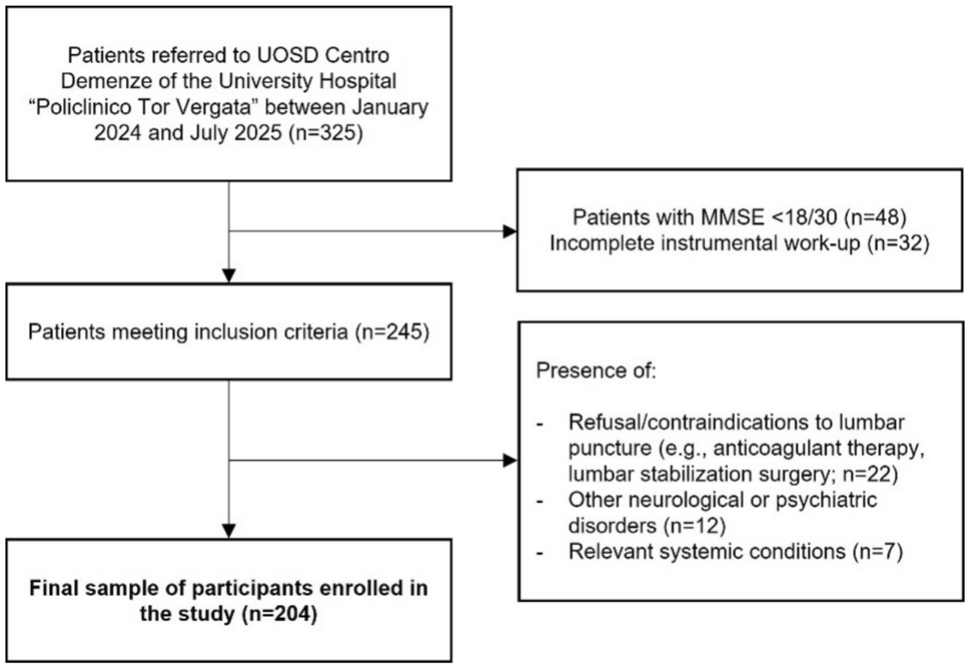
Flowchart summarizing the enrollment procedures for the present study. *MMSE* Mini-Mental State Examination

Exclusion criteria were: (1) refusal to undergo lumbar puncture; (2) diagnosis of other neurological or psychiatric disorders; (3) anatomical or pharmacological contraindications to lumbar puncture (e.g., oral anticoagulant therapy, previous lumbar stabilization surgery); (4) alternative systemic or metabolic conditions that could account for cognitive decline. Enrollment procedures are summarized in Fig. 1.

### CSF, blood sampling, and biomarkers analysis

All lumbar punctures were performed between 8 and 10 am. CSF samples of around 8 mL were collected in polypropylene tubes and processed according to laboratory standard operating procedures. 2 mL were used for biochemical routine analysis, including cell and protein count, while 6 mL were centrifuged at 2000 g at + 4 °C for 10 min, aliquoted in 1 mL portions and frozen at − 80 °C for further analysis. Blood samples were collected at ± 30 min from lumbar punctures and centrifuged at 3500 g at + 4 °C for 10 min. Plasma was then aliquoted and frozen with the same procedures used for CSF samples. Blood samples were subjected to routine laboratory testing, including measurement of serum creatinine levels and estimated Glomerular Filtration Rate (eGFR).

CSF levels of Aβ40, Aβ42, p-tau181, and total tau, as well as plasma levels of Aβ40, Aβ42, p-tau181, and p-tau217, were measured using fully automated CLEIA Fujirebio LUMIPULSE^®^ G1200 (Fujirebio, Inc., Tokyo, Japan). In accordance with the NIA-AA revised criteria, we defined the presence of AD pathological changes based on alterations in CSF “core 1” biomarkers [5]. Positivity was defined by an abnormality in CSF p-tau181/Aβ42 ratio (pathological value ≥ 0.069), derived from previous internal validation and consistent with reference values reported in the literature [17]. Moreover, we computed the plasma p-tau181/Aβ42 and p-tau217/Aβ42 ratio.

### Statistical analysis

After classifying the study population according to the presence (AD) or absence (non-AD) of at least one abnormal “core 1” CSF biomarker, we assessed differences between the two subgroups via Mann–Whitney U test for continuous variables and with Chi-squared test for categorical variables. Subsequently, using receiver operating characteristic (ROC) analysis, we evaluated the performance of plasma biomarkers (p-tau181, p-tau217 and the p-tau181/Aβ42 and p-tau217/Aβ42 ratios) in discriminating patients classified as AD from non-AD. Comparison of the respective areas under the curve (AUC) was performed using DeLong’s test. To determine optimal cut-offs, we used Youden’s index (J = sensitivity + specificity − 1). Given the potential risk of biological coupling between the CSF reference for AD pathology and the homologous plasma biomarker ratio, we performed an additional sensitivity analysis using an alternative outcome. In this analysis, subjects were classified as A + T + in the presence of both abnormal CSF Aβ42/40 (< 0.0667) and CSF p-tau181 (> 56 pg/mL), while the remaining subjects were classified as non-A + T +. ROC analyses were repeated to test the performance of plasma p-tau181, plasma p-tau181/Aβ42, plasma p-tau217, and plasma p-tau217/Aβ42, AUCs were compared using DeLong’s test.

We then derived each patient’s global predicted probability of AD pathology (P_core1+_) from the individual plasma biomarker values, according to the criteria described above. For this purpose, we used the sensitivity and specificity values obtained from the ROC analyses, together with the prevalence of the disease in our cohort, according to the following formula:

$${P}_{core1+}=Sensitivity\times Prevalence+(1-Specificity)\times (1-Prevalence)$$

We subsequently applied the two-step approach [7] stratifying patients into three risk categories (high, intermediate, low). Risk categories were defined using 90% thresholds: the high-risk threshold was set at 90% specificity and the low-risk threshold at 90% sensitivity. The overall accuracy of the two-step approach was calculated as the proportion of correctly classified cases in the high- and low-risk groups, according to the following formula:

$$Accuracy= \frac{True Positives+True Negatives}{{n}_{high risk}+ {n}_{low risk}}$$

For each biomarker, the two-cut-off strategy was compared with a single-threshold approach with respect to the following parameters: accuracy, positive predictive value (PPV), and negative predictive value (NPV), with their corresponding 95% confidence intervals (95% CI), calculated using Clopper–Pearson analysis.

Subsequently, we performed subgroup analyses, estimating the accuracy with 95% CI of individual biomarkers after stratification by the following variables: sex (male/female); age (< 75/≥ 75 years); and presence or absence of chronic kidney disease (CKD −/CKD +). The latter was defined as eGFR < 60 ml/min/1.73 m^2^, calculated using the CKD-EPI equation [18].

We further stratified the cohort into two groups according to the degree of cognitive decline based on the patients’ median estimated MMSE score (26/30) and assessed the diagnostic performance of plasma biomarkers using ROC curves. Comparison of the respective AUC values was carried out with DeLong’s test, in order to identify any significant differences in the discriminatory ability of the biomarkers according to clinical stage.

Statistical analysis and data management were operated via jamovi (jamovi, version 2.4; software available at https://www.jamovi.org). Images were generated with GraphPad Prism^©^ version 10.1.0 for Windows (GraphPad Software, San Diego, California USA, www.graphpad.com).

All results were computed with two-tailed significance tests; values of *p* < 0.05 were considered statistically significant.

## Results

The study included 204 patients. Demographic, clinical, and laboratory characteristics of the study population are reported as medians and interquartile range for non-normally distributed data (Table 1).

**Table 1:** Demographic, clinical, and laboratory characteristics of the study population, stratified according to CSF-based diagnosis of AD vs non-AD

	AD (*n* = 113)	Non-AD (*n* = 91)	Test statistics (*U*)	*p*
Age	76.02 (7.52)	72.25 (10.66)	3768.5	0.001***
Sex (M:F)	49:64	63:28	n.a	< 0.001***
MMSE	24.00 (6.27)	27.50 (3.00)	7174.0	< 0.001***
CSF Aß42/Aß40	0.049 (0.010)	0.100 (0.010)	3901.0	< 0.001***
CSF p-tau181 (pg/ml)	87.20 (55.10)	28.60 (16.50)	504.5	< 0.001***
CSF t-tau (pg/ml)	577.00 (321.00)	235.00 (124.30)	690.0	< 0.001***
CSF p-tau181/Aß42	0.184 (0.140)	0.031 (0.017)	0.0	< 0.001***
Plasma Aß42/Aß40	0.080 (0.010)	0.096 (0.017)	7538.0	< 0.001***
Plasma p-tau181 (pg/ml)	2.23 (1.14)	1.12 (0.69)	1360.5	< 0.001***
Plasma p-tau181/Aß42	0.083 (0.041)	0.033 (0.020)	734.0	< 0.001***
Plasma p-tau217 (pg/ml)	0.411 (0.440)	0.087 (0.093)	1199.5	< 0.001***
Plasma p-tau217/Aß42	0.015 (0.019)	0.003 (0.003)	1075.5	< 0.001***
eGFR (ml/min/1.73 m2)	83.55 (17.22)	86.26 (22.96)	5119.0	0.639

Data are presented as median (interquartile range) or proportions, when applicable

*AD* Alzheimer’s disease, *CSF* cerebrospinal fluid, *p-tau* phosphorylated tau, *t-tau* total tau, *MMSE* Mini-Mental State Examination, *eGFR* estimated Glomerular Filtration Rate. Bold means p<0.05.

***p ≤ 0.001

### ROC analysis of plasma biomarkers

**Fig. 2:**
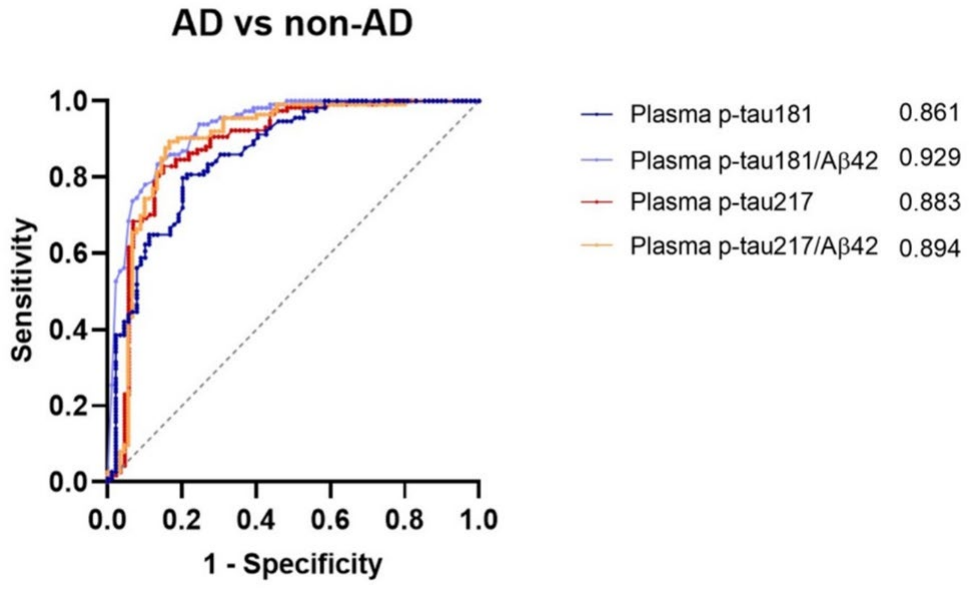
ROC curves of the analyzed plasma biomarkers (p-tau181, p-tau181/Aβ42, p-tau217, p-tau217/Aβ42) showing their performance in discriminating AD cases—defined by the presence of at least one abnormal “core 1” biomarker—from non-AD individuals. The corresponding AUC (area under the curve) values are shown to the right of each biomarker. *AD* Alzheimer’s disease, *p-tau* phosphorylated tau

We performed a ROC analysis to evaluate the performance of plasma biomarkers in predicting AD (Fig. 2). The optimal cut-offs for each biomarker, identified using Youden’s index, were as follows: plasma p-tau181 ≥ 1.58 pg/mL, plasma p-tau181/Aβ42 ≥ 0.054, plasma p-tau217 ≥ 0.207 pg/ml, plasma p-tau217/Aβ42 ≥ 0.006. Among individual biomarkers, the highest AUC was observed for plasma p-tau217 (AUC 0.883; 95% CI 0.830–0.937), followed by plasma p-tau181 (AUC 0.866; 95% CI 0.816–0.917). The plasma p-tau181/Aβ42 ratio showed better overall performance (AUC 0.928; 95% CI 0.891–0.965) with respect to p-tau217/Aβ42 ratio (AUC 0.894; 95% CI 0.843–0.945) and individual p-tau biomarkers, but comparison of the AUC values using DeLong’s test revealed a statistically significant difference only between p-tau181/Aβ42 ratio and p-tau181 (*p* < 0.001). Consistent with the main analysis, in the sensitivity analysis based on the A + T + versus non-A + T + definition, plasma p-tau181/Aβ42 showed the best performance (AUC 0.901; 95% CI 0.859–0.943), followed by plasma p-tau217/Aβ42 (AUC 0.873; 95% CI 0.827–0.925), plasma p-tau217 (AUC 0.872; 95% CI 0.822–0.922), and plasma p-tau181 (AUC 0.857; 95% CI 0.807–0.909) (Supplementary material—Fig. S1). DeLong’s test revealed a statistically significant difference only between plasma p-tau181/Aβ42 and plasma p-tau181 (*p* = 0.015).

### Application of the two-cut-off model

**Fig. 3:**
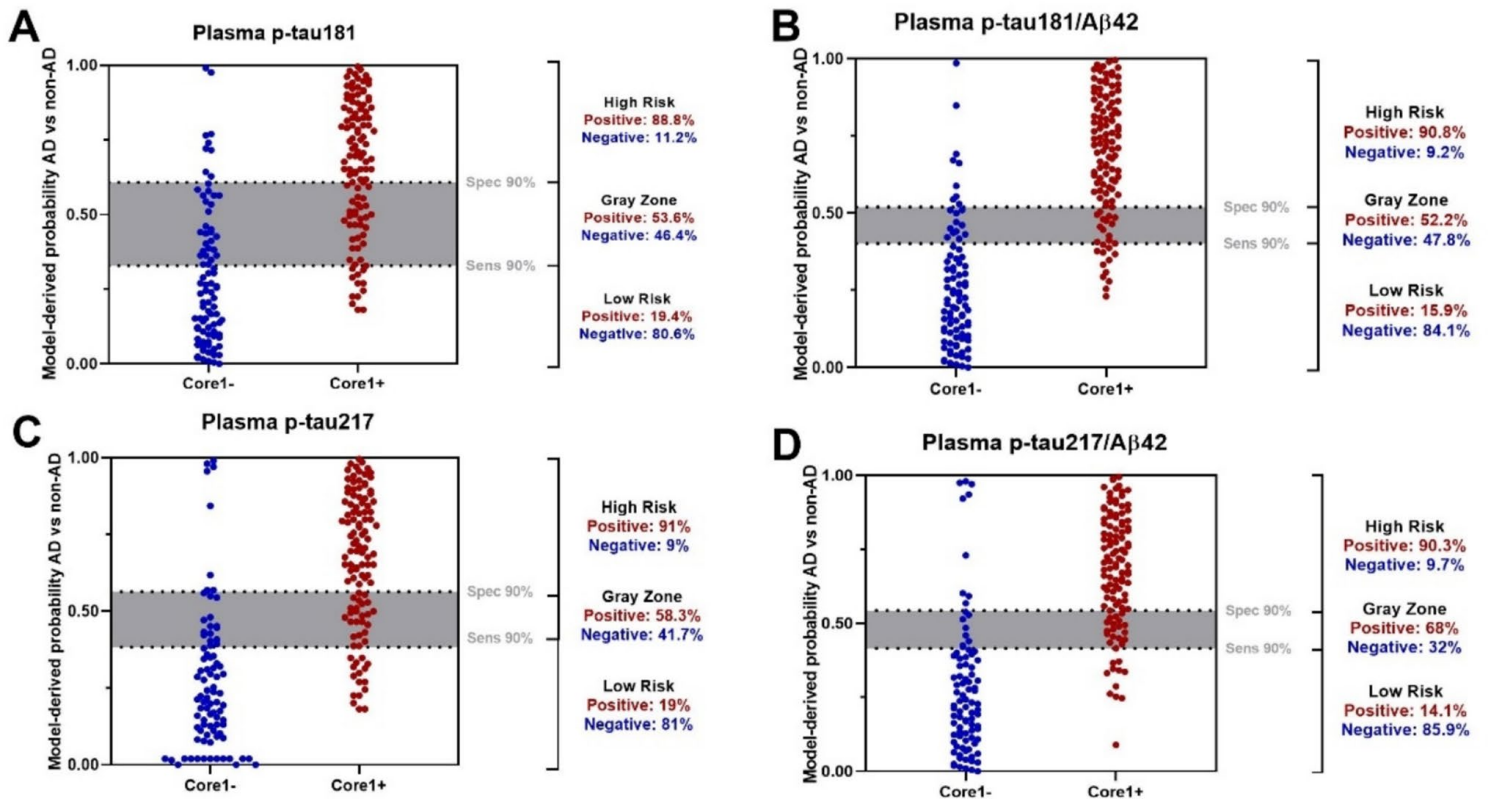
Model-derived probabilities for AD vs non-AD classification obtained by applying the two-cut-off approach (90% sensitivity, 90% specificity) for each plasma biomarker: **a** p-tau181, **b** p-tau181/Aβ42, **c** p-tau217, **d** p-tau217/Aβ42. Each dot represents an individual; subjects with ≥ 1 abnormal CSF “core 1” biomarker (core1 +) are shown in red, and those without abnormalities (core1 −) in blue. Dashed lines indicate the cut-off thresholds; the grey band represents the intermediate zone. The proportions of patients in the low-, intermediate-, and high-risk categories are shown to the right of each panel. *AD* Alzheimer’s disease, *p-tau* phosphorylated tau

Patients were further classified into high-, intermediate-, and low-risk categories based on the probabilities derived from individual plasma biomarkers values, using a two-cut-off model with thresholds set at 90% specificity and sensitivity (Fig. 3).

**Fig. 4:**
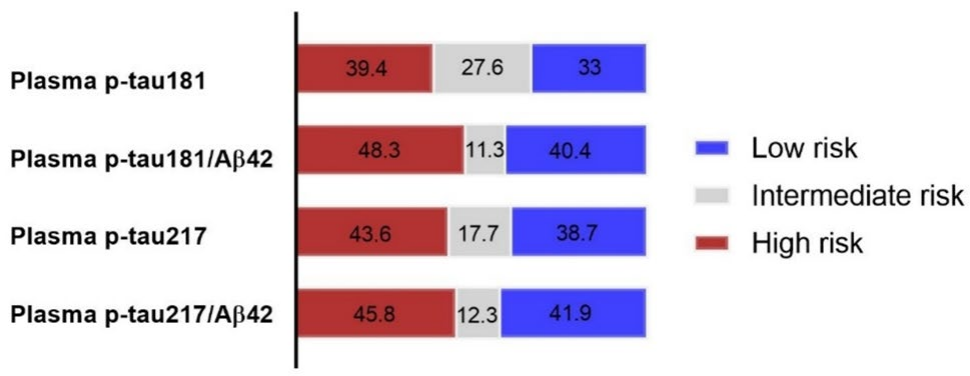
The figure shows the distribution of participants across low-, intermediate- and high-risk categories for Alzheimer’s disease for each plasma biomarker. Bars represent 100% of the cohort and percentages are reported within each segment. *p-tau* phosphorylated tau

The proportion of patients within each risk category (%) is shown in Fig. 4. According to the recommendations from the Global CEO Initiative on AD, a BBB test should classify no more than 15–20% of individuals as intermediate in a typical clinical population, so we considered 20% as a reference threshold for acceptable performance [19]. Overall, the p-tau181/Aβ42 and ptau217/Aβ42 ratios showed the smallest intermediate zone (11.3 and 12.3%, respectively) and the highest proportion of deterministic classifications (low-risk and high-risk), suggesting superior discriminative ability between AD and non-AD. Plasma p-tau217 maintained a proportion of intermediate classifications below the reference threshold (17.7%), while plasma p-tau181 displayed the highest rate of indeterminate classifications (27.6%) and the lowest proportion of classifications falling into either the high- or low-risk category.

### Single and two-cut-off models: comparison of diagnostic accuracy

Using the two-cut-off strategy, overall diagnostic accuracy and notably rule-in ability (PPV) improved for all plasma biomarkers.

For plasma p-tau181, accuracy increased from 0.803 (95% CI 0.742–0.855) with a single cut-off to 0.850 (95% CI 0.782–0.904) with the two-cut-off approach; PPV rose from 0.835 (0.768–0.885) to 0.888 (0.797–0.947), and NPV from 0.766 (0.689–0.828) to 0.806 (0.691–0.892). A similar pattern was observed for the p-tau181/Aβ42 ratio: accuracy increased from 0.852 (0.796–0.898) to 0.878 (0.821–0.922), with PPV rising from 0.888 (0.823–0.931) to 0.908 (0.833–0.957) and NPV from 0.813 (95% CI 0.738–0.869) to 0.841 (0.744–0.913).

Likewise, the two-cut-off model also improved the performance of plasma p-tau217: accuracy increased from 0.848 (0.791–0.894) to 0.863 (0.802–0.911), PPV from 0.873 (0.808–0.918) to 0.910 (0.831–0.960), and NPV from 0.819 (0.743–0.876) to 0.840 (0.750–0.908). Finally, for p-tau217/Aβ42, accuracy increased from 0.867 (0.812–0.911) to 0.882 (0.825–0.925); PPV from 0.871 (0.809–0.915) to 0.903 (0.824–0.955), while NPV remained virtually unchanged, from 0.862 (0.784–0.915) to 0.859 (0.766–0.925).

**Fig. 5:**
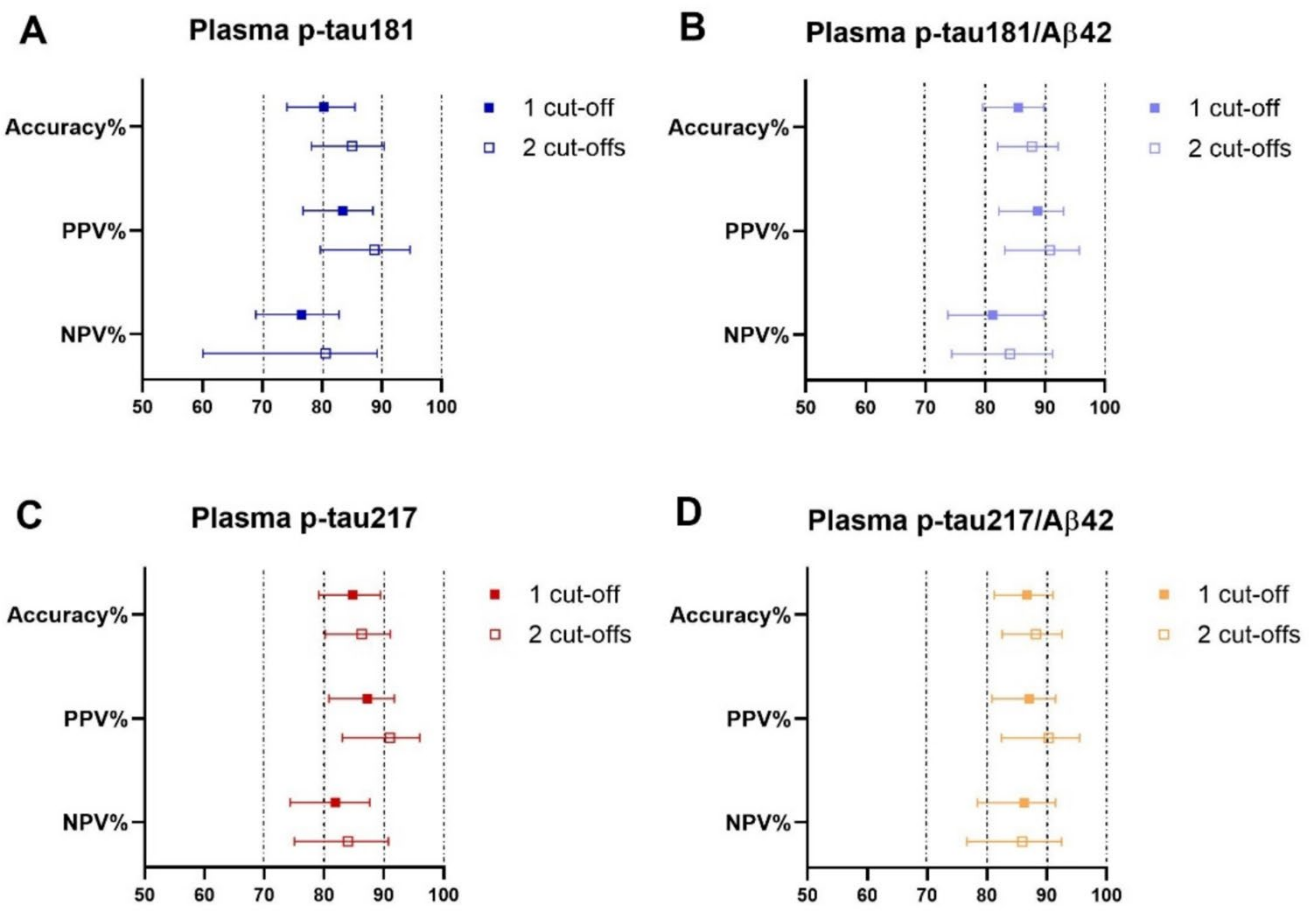
Forest plots comparing the single-cut-off model and the two-cut-off model (90% sensitivity/90% specificity) for the analyzed plasma biomarkers: **a** p-tau181, **b** p-tau181/Aβ42, **c** p-tau217, **d** p-tau217/Aβ42. Each panel reports accuracy, positive predictive value (PPV), and negative predictive value (NPV) with 95% CI. Squares represent estimates obtained with the single-cut-off, circles those obtained with the two-cut-off. In the dual-threshold strategy, accuracy is calculated only on deterministic cases (low- and high-risk). *PPV* positive predictive value, *NPV* negative predictive value, *p-tau* phosphorylated tau

Overall, the two-cut-off strategy enhanced diagnostic precision at both extremes of risk, with more pronounced gains in PPV. Among the biomarkers, p-tau181/Aβ42 and p-tau217/Aβ42 retained the highest and most stable estimates (Fig. 5).

### Diagnostic accuracy across subgroups stratified by sex, age, and renal function

Stratifying participants into subgroups according to sex (male/female), age (< 75/≥ 75 years), presence or absence of chronic kidney disease (CKD −/CKD +), and diagnostic accuracy remained generally high and showed consistent patterns across strata.

With respect to sex, accuracy estimates were systematically higher in women than in men, with the most pronounced differences observed for p-tau217-based biomarkers: plasma p-tau217 92.39% (95% CI 84.95–96.89) vs 79.46% (95% CI 70.80–86.51); p-tau217/Aβ42 94.41% (95% CI 86.20–97.54) vs 81.25% (95% CI 72.78–88.00); p-tau181/Aβ42 94.51% (95% CI 87.64–98.19) vs 85.71% (95% CI 77.84–91.61); p-tau181 87.91% (95% CI 79.40–93.81) vs 83.04% (95% CI 74.78–89.46).

Regarding age, a decline in accuracy was observed in participants aged ≥ 75 years compared with those < 75 years: for plasma p-tau181, accuracy decreased from 87.04% (95% CI 79.21–92.73) to 70.53% (95% CI 60.29–79.44); for p-tau181/Aβ42 from 89.81% (95% CI 82.51–94.80) to 83.16% (95% CI 74.10–90.06); for p-tau217 from 88.99% (95% CI 81.56–94.18) to 81.05% (95% CI 71.72–88.37); for p-tau217/Aβ42 from 87.96% (95% CI 80.30–93.43) to 84.21% (95% CI 75.30–90.88).

When stratifying by renal function, accuracy estimates in CKD − participants ranged between approximately 82% and 87% (p-tau181 81.56%, 95% CI 75.10–86.96; p-tau181/Aβ42 87.15%, 95% CI 81.35–91.68; p-tau217 85.00%, 95% CI 78.93–89.88; p-tau217/Aβ42 86.03%, 95% CI 80.08–90.75). In CKD + participants, confidence intervals were wider—likely reflecting the smaller subgroup size—yet the performance of p-tau217-based markers remained high (90.00%, 95% CI 68.30–98.77), compared with p-tau181 (75.00%, 95% CI 50.90—91.34).

**Fig. 6:**
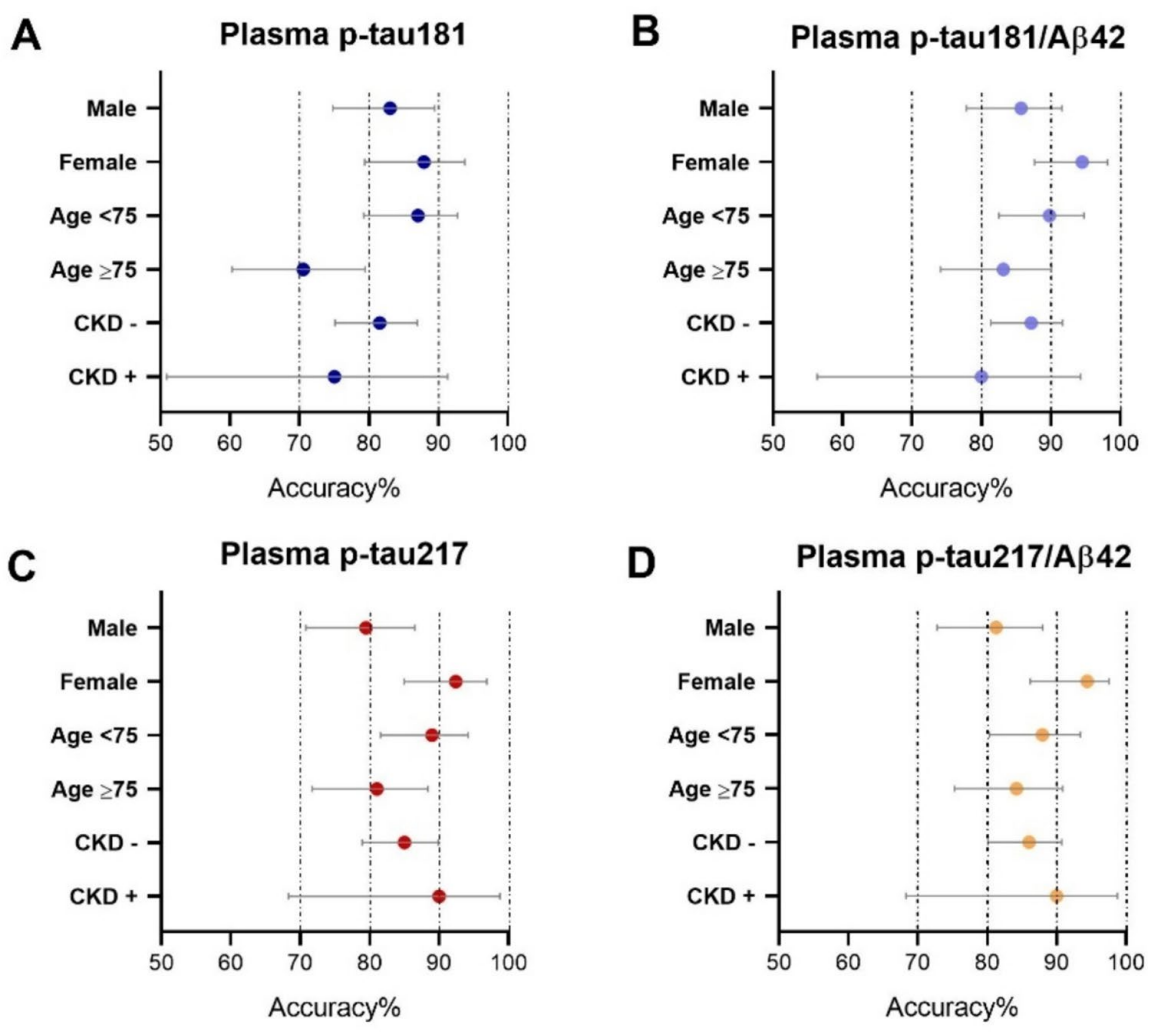
Forest plots illustrating the accuracy of plasma biomarkers, with participants stratified into subgroups according to sex (male/female), age (< 75/≥ 75 years), and presence or absence of chronic kidney disease (CKD +/−). Filled and open symbols identify the two classes within each subgroup; horizontal bars indicate 95% confidence intervals (95% CI). **a** p-tau181, **b** p-tau181/Aβ42, **c** p-tau217, **d** p-tau217/Aβ42. *CKD* chronic kidney disease, *p-tau* phosphorylated tau

Overall, Aβ42-adjusted ratios—particularly p-tau181/Aβ42—yielded high and stable accuracy estimates across stratifications. Advanced age and CKD were associated with reduced precision (wider confidence intervals) and, in the case of age, with a measurable decrease in diagnostic accuracy (Fig. 6).

### Diagnostic accuracy across subgroups stratified by cognitive impairment

To assess the impact of cognitive decline on the discriminative ability of plasma biomarkers, ROC analyses were performed separately according to MMSE score (≥ 26/30 vs < 26/30).

In subjects with MMSE ≥ 26/30, the best performance was observed for the hybrid ratios: p-tau181/Aβ42 reached an AUC of 0.921 (95% CI 0.868–0.974), followed by p-tau217/Aβ42 with an AUC of 0.901 (95% CI 0.836–0.966). Among single analytes, p-tau217 achieved an AUC of 0.882 (95% CI 0.810–0.953), whereas p-tau181 reached 0.836 (95% CI 0.754–0.919).

In subjects with MMSE < 26/30, p-tau181/Aβ42 showed an AUC of 0.903 (95% CI 0.821–0.984) and p-tau181 an AUC of 0.860 (95% CI 0.773–0.948), while p-tau217-based biomarkers displayed lower discriminative ability (p-tau217/Aβ42: 0.842, 95% CI 0.738–0.945; p-tau217: 0.824, 95% CI 0.710–0.938).

**Fig. 7:**
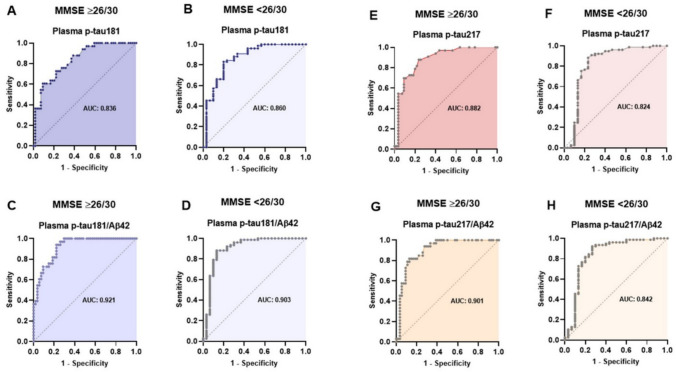
ROC curves of plasma biomarkers stratified by MMSE (≥ 26/30 vs < 26/30). The corresponding AUC values are reported within each plot. **a**, **b** p-tau181, **c**, **d** p-tau181/Aβ42, **e**, **f** p-tau217, **g**, **h** p-tau217/Aβ42. *AUC* area under the curve, *MMSE *Mini-Mental State Examination, *p-tau* phosphorylated tau

Overall, Aβ42-adjusted ratios yielded higher AUC in both groups. Comparison of the AUC values using DeLong’s test confirmed a statistically significant difference only between plasma p-tau181/Aβ42 and p-tau181 both in subjects with MMSE ≥ 26/30 (*p* = 0.012) and in those with MMSE < 26/30 (*p* = 0.033). The full results are presented in Fig. 7.

## Discussion

Risk-stratification models based on plasma biomarkers have been proposed as first-line screening tools for patients with suspected AD, with second-line investigations reserved for uncertain cases [7]. In our single-center cohort, we applied a two-cut-off approach previously validated in multicenter studies, to assess predictive performance of plasma biomarkers both as individual analytes (p-tau181 and p-tau217) and as ratios normalized to Aβ42 (p-tau181/Aβ42, p-tau217/Aβ42) in clinical practice.

Adoption of the two-cut-off strategy yielded superior discriminative performance compared with a single-cut-off model: in the low- and high-risk groups, we observed higher overall accuracy and improved rule-in capacity, defined by higher PPV. In practical terms, using the two-cut-off approach, a substantial proportion of patients could have completed the diagnostic work-up at this initial stage of the workflow, without requiring additional confirmatory testing. Nonetheless, a “gray zone” of intermediate risk remains, in which plasma results are inconclusive relative to CSF-based classification; these patients, whose plasma biomarkers values represent an area of overlap between AD and non-AD, require a second diagnostic step (e.g., CSF or PET) to establish a definitive diagnosis. In our cohort, Aβ42-normalized ratios substantially reduced the width of the intermediate zone: for p-tau181/Aβ42 and p-tau217/Aβ42, the proportion of indeterminate classifications falls to 11–12%, below the ideally recommended 15–20% threshold [19].

Our findings are consistent with the recent literature. Specifically, a two-cut-off approach leveraging the p-tau217/Aβ42 ratio yielded an accuracy between 91%−94% across different single-center and multicenter cohorts [9, 20, 21]. In our study, accuracy estimates were slightly lower, yet remaining close to the recommended benchmark of 90% sensitivity and specificity indicated by the Global CEO initiative on AD [19], which also highlight that it may be acceptable to use BBBs test with lower specificity (≥ 75–85%) in secondary care settings, particularly when applying the two-threshold approach to p-tau/Aβ42 ratios.

Our analysis also showed different accuracy of plasma biomarkers when considering the effect of specific demographic and clinical variables. Stratification by sex exhibited higher accuracy in women than in men, with particularly marked differences for p-tau217-based biomarkers. When stratifying by age, we observed lower diagnostic accuracy among participants aged ≥ 75 years, consistent with previous reports [20]. In subjects with CKD, diagnostic precision was overall lower, with wider confidence intervals partly reflecting the smaller subgroup size, although p-tau217 and p-tau217/Aβ42 showed slightly better accuracy than p-tau181-based biomarkers. From a biological perspective, these differences may reflect distinct interactions between plasma tau biomarkers and patient-specific factors such as sex, age, and comorbidity burden. The reduced accuracy observed in older participants may reflect greater clinical heterogeneity, lower AD burden, and a higher prevalence of co-pathologies [23, 24], whereas previous evidence has suggested higher tau accumulation in women and in younger Aβ-positive individuals [22]. Taken together, these findings may suggest accounting for demographic profile and comorbidities when making diagnostic decisions based on plasma biomarkers in clinical practice.

Moreover, Aβ42-normalized ratios seem to offer greater robustness to such modifying factors, since they perform systematically better than their stand-alone counterparts, even when considering patients in different stages of cognitive decline. Both plasma p-tau181/Aβ42 and p-tau217/Aβ42 exhibited better discriminative ability in earlier clinical stages, in line with previously published data [9]. Indeed, normalization to Aβ42 enhances discriminative performance, consistent with the evidence that Aβ42 has greater diagnostic utility in the early rather than the advanced stages of the disease [25]. In our study cohort, p-tau181-based biomarkers showed relatively better performances in more advanced clinical stages, with p-tau181/Aβ42 having the highest accuracy. One possible explanation is that p-tau217 might be more sensitive in earlier phases of the biological continuum [26, 27]. This seems to prompt the implementation of a dynamic and adaptive strategy, where hybrid ratio should be systematically preferred to stand-alone biomarkers, and in which the choice of p-tau species might need to be tailored to the clinical context.

The optimal setting for the clinical implementation of plasma biomarkers remains to be fully defined. At present, their use appears most realistic and appropriate in specialist centers, where established clinical and imaging diagnostic pathways can be readily available upon clinical judgement. We believe that evaluating the real-world performance of local laboratory workflows holds an important place to both better interpret biomarker results and to optimize referral pathways, positioning specialist centers as effective reference hubs for primary care and community-based services. More broadly, additional studies are needed to define implementation strategies, to validate plasma biomarker performance in heterogeneous populations, and to assess cost-effectiveness of referral in real-world settings.

This study has both strengths and limitations. A key strength is the consecutive inclusion of outpatients undergoing lumbar puncture and blood sampling for AD biomarkers over an 18-month period at our Memory Clinic, which minimizes selection bias. In addition, the consistency of analytical procedures for CSF and plasma, and the use of internally derived cut-offs aligned with the literature, strengthen the reliability of our estimates. Among the limitations, the fact that plasma biomarker cut-offs were derived and tested in the same cohort entails an intrinsic risk of overfitting, while the retrospective and single-center design may limit the generalizability of our findings. This study was conducted in a specialist memory clinic population, characterized by a relatively high pre-test probability of AD, and the observed diagnostic performance may therefore not directly translate to less selected or broader clinical settings. In addition, some subgroups (e.g., CKD +) were relatively small, resulting in wider confidence intervals, and the dependence of PPV and NPV on disease prevalence requires caution when extrapolating these estimates to populations with different baseline risk. An additional limitation concerns the primary CSF reference standard, which may have introduced at least partial methodological alignment with the homologous plasma p-tau181/Aβ42 ratio. Although a sensitivity analysis based on the alternative A + T + definition showed results that were overall consistent with the main analysis, our finding should be interpreted cautiously and requires confirmation in independent studies.

## Conclusion

Our findings support the two-step diagnostic model using plasma p-tau ratios (p-tau181/Aβ42 or p-tau217/Aβ42) to identify low- and high-risk cases of AD in an unselected real-world memory clinic cohort. This workflow can streamline and accelerate diagnosis, while maintaining appropriate selectivity for therapeutic pathways that require confirmation of amyloid pathology. Demographic profiles and comorbidities should be considered when interpreting plasma biomarker results, as they may influence absolute concentrations of the analytes and the applicability of predefined cut-offs.

At present, the use of plasma-based tests appears feasible within specialist centers, where plasma assays can support and complement established diagnostic pathways based on clinical evaluation and CSF/neuroimaging biomarkers. Given the considerable variability of accuracy, particularly across demographic factors such as age and sex, their implementation in primary care remains challenging. Indeed, an accurate interpretation of test results still relies on specialist expertise and integration with established diagnostic pathways. Looking ahead, pre-analytical and analytical standardization together with local refinement of cut-offs may allow these biomarkers to function as reliable triage tools, shifting diagnosis from invasive procedures to minimally invasive tests, shortening time to diagnosis, and optimizing resource allocation. This would represent a concrete step toward scalable precision-medicine pathways, which is particularly relevant in the present rapidly evolving therapeutic landscape.

## Supplementary Information

Below is the link to the electronic supplementary material.

Supplementary file1 (DOCX 111 KB)

## Data Availability

The data supporting the findings of this study is available on request from the corresponding author. The data is not publicly available due to privacy or ethical restrictions.

## References

[1] Jack CR, Bennett DA, Blennow K et al (2018) NIA-AA research framework: toward a biological definition of Alzheimer’s disease. Alzheimer’s Dement 14:535–562 29653606 10.1016/j.jalz.2018.02.018 PMC5958625

[2] Shaw LM, Arias J, Blennow K et al (2018) Appropriate use criteria for lumbar puncture and cerebrospinal fluid testing in the diagnosis of Alzheimer’s disease. Alzheimer’s Dement 14:1505–1521 30316776 10.1016/j.jalz.2018.07.220 PMC10013957

[3] Leuzy A, Mattsson-Carlgren N, Cullen NC et al (2023) Robustness of CSF Aβ42/40 and Aβ42/P-tau181 measured using fully automated immunoassays to detect AD-related outcomes. Alzheimers Dement 19:2994–3004 36681387 10.1002/alz.12897

[4] Schindler SE, Atri A (2023) The role of cerebrospinal fluid and other biomarker modalities in the Alzheimer’s disease diagnostic revolution. Nat Aging 3:460 37202514 10.1038/s43587-023-00400-6 PMC10720501

[5] Jack CR, Andrews JS, Beach TG et al (2024) Revised criteria for diagnosis and staging of Alzheimer’s disease: Alzheimer’s Association Workgroup. Alzheimer’s Dement 20:5143–5169 38934362 10.1002/alz.13859 PMC11350039

[6] Ashton NJ, Brum WS, Di MG et al (2024) Diagnostic accuracy of a plasma phosphorylated tau 217 immunoassay for Alzheimer disease pathology. JAMA Neurol 81:255–263 38252443 10.1001/jamaneurol.2023.5319 PMC10804282

[7] Brum WS, Cullen NC, Janelidze S et al (2023) A two-step workflow based on plasma p-tau217 to screen for amyloid β positivity with further confirmatory testing only in uncertain cases. Nat Aging 3:1079–1090 37653254 10.1038/s43587-023-00471-5 PMC10501903

[8] Mielke MM, Anderson M, Ashford JW et al (2024) Recommendations for clinical implementation of blood-based biomarkers for Alzheimer’s disease. Alzheimers Dement 20:8216–8224 39351838 10.1002/alz.14184 PMC11567872

[9] Sylvain Lehmann A, Gabelle A, Duchiron M, et al. The ratio of plasma pTau217 to Aβ42 outperforms individual measurements in detecting brain amyloidosis. 10.1101/2024.12.07.24318640

[10] Ashton NJ, Puig-Pijoan A, Milà-Alomà M et al (2023) Plasma and CSF biomarkers in a memory clinic: head-to-head comparison of phosphorylated tau immunoassays. Alzheimer’s Dement 19:1913–1924 36370462 10.1002/alz.12841 PMC10762642

[11] Parvizi T, Wurm R, König T et al (2024) Real-world performance of plasma p-tau181 in a heterogeneous memory clinic cohort. Ann Clin Transl Neurol 11:1988–1998 38965832 10.1002/acn3.52116 PMC11330220

[12] Arranz J, Zhu N, Rubio-Guerra S et al (2024) Diagnostic performance of plasma pTau217, pTau181, Aβ1–42 and Aβ1–40 in the LUMIPULSE automated platform for the detection of Alzheimer disease. Alzheimers Res Ther. 10.1186/s13195-024-01513-9 38926773 10.1186/s13195-024-01513-9 PMC11200993

[13] Pichet Binette A, Janelidze S, Cullen N et al (2023) Confounding factors of Alzheimer’s disease plasma biomarkers and their impact on clinical performance. Alzheimer’s Dement 19:1403–1414 36152307 10.1002/alz.12787 PMC10499000

[14] Carlesimo GA, Caltagirone C, Gainotti G et al (1996) The mental deterioration battery: normative data, diagnostic reliability and qualitative analyses of cognitive impairment. Eur Neurol 36:378–384 8954307 10.1159/000117297

[15] McKhann GM, Knopman DS, Chertkow H et al (2011) The diagnosis of dementia due to Alzheimer’s disease: recommendations from the National Institute on Aging-Alzheimer’s Association workgroups on diagnostic guidelines for Alzheimer’s disease. Alzheimer’s Dement 7:263–269 21514250 10.1016/j.jalz.2011.03.005 PMC3312024

[16] Albert MS, Dekosky ST, Dickson D et al (2011) The diagnosis of mild cognitive impairment due to Alzheimer’disease: recommendations from the National Institute on Aging-Alzheimer’s Association workgroups on diagnostic guidelines for Alzheimer’s disease. Alzheimer’s Dement 7:270–279 21514249 10.1016/j.jalz.2011.03.008 PMC3312027

[17] Campbell MR, Ashrafzadeh-Kian S, Petersen RC et al (2021) P-tau/Aβ42 and Aβ42/40 ratios in CSF are equally predictive of amyloid PET status. Alzheimer’s Dement. 10.1002/dad2.12190 10.1002/dad2.12190 PMC8129859 34027020

[18] Levey AS, Stevens LA, Schmid CH et al (2009) A new equation to estimate glomerular filtration rate. Ann Intern Med 150:604 19414839 10.7326/0003-4819-150-9-200905050-00006 PMC2763564

[19] Schindler SE, Galasko D, Pereira AC et al (2024) Acceptable performance of blood biomarker tests of amyloid pathology—recommendations from the Global CEO Initiative on Alzheimer’s disease. Nat Rev Neurol 20:426–439 38866966 10.1038/s41582-024-00977-5

[20] Palmqvist S, Warmenhoven N, Anastasi F et al (2025) Plasma phospho-tau217 for Alzheimer’s disease diagnosis in primary and secondary care using a fully automated platform. Nat Med 31:2036 40205199 10.1038/s41591-025-03622-w PMC12176611

[21] Giacomucci G, Crucitti C, Ingannato A et al (2025) The two cut-offs approach for plasma p-tau217 in detecting Alzheimer’s disease in subjective cognitive decline and mild cognitive impairment. Alzheimers Dement (Amst). 10.1002/DAD2.70116 40357140 10.1002/dad2.70116 PMC12066393

[22] Smith R, Strandberg O, Mattsson-Carlgren N et al (2020) The accumulation rate of tau aggregates is higher in females and younger amyloid-positive subjects. Brain 143:3805–3815 33439987 10.1093/brain/awaa327 PMC7805812

[23] Schöll M, Ossenkoppele R, Strandberg O et al (2017) Distinct 18F-AV-1451 tau PET retention patterns in early- and late-onset Alzheimer’s disease. Brain 140:2286–2294 29050382 10.1093/brain/awx171

[24] Bonomi CG, Motta C, Di Donna MG et al (2025) Rethinking dementia in the oldest old: lessons to learn for the diagnosis and treatment of Alzheimer’s disease. Neural Regen Res. 10.4103/NRR.NRR-D-25-00312 40637583 10.4103/NRR.NRR-D-25-00312 PMC13378931

[25] Albani D, Marizzoni M, Ferrari C et al (2019) Plasma Aβ42 as a biomarker of prodromal Alzheimer’s disease progression in patients with amnestic mild cognitive impairment: evidence from the PharmaCog/E-ADNI study. J Alzheimers Dis 69:37–48 30149449 10.3233/JAD-180321

[26] Janelidze S, Berron D, Smith R et al (2021) Associations of plasma phospho-tau217 levels with tau positron emission tomography in early Alzheimer’s disease. JAMA Neurol 78:149–156 33165506 10.1001/jamaneurol.2020.4201 PMC7653537

[27] Yakoub Y, Gonzalez-Ortiz F, Ashton NJ et al (2025) Plasma p-tau217 identifies cognitively normal older adults who will develop cognitive impairment in a 10-year window. Alzheimers Dement. 10.1002/ALZ.14537 40008832 10.1002/alz.14537 PMC11863240

