# Supplementary material for: Streamlining Alzheimer’s disease diagnosis: real-world validation of two-cut-off diagnostic models based on plasma p-tau/Aβ42 ratios
Source: J Neurol. 2026 May 8;273(6):306. doi: 10.1007/s00415-026-13833-x (PMC13156155; doi:10.1007/s00415-026-13833-x)
Supplement: Supplementary file 1 — Supplementary file1 (DOCX 111 KB) [file 415_2026_13833_MOESM1_ESM.docx]

*Original Communication – Journal of Neurology*

**Streamlining Alzheimer’s disease diagnosis: real-world validation of two-cut-off diagnostic models based on plasma p-tau/Aβ42 ratios**

Martina Poli^1,2^*, Chiara Giuseppina Bonomi^1^*, Martina Gaia Di Donna^1^, Ilaria Barberis^1^, Emanuele Luca Ginevra^1^, Marzia Nuccetelli^3^, Sergio Bernardini^3^, Diego Centonze^2,4^, Alessandro Martorana^1,2^, Caterina Motta^1^

^1^Memory Clinic and Neurodegenerative Dementia Research Unit, Policlinico Tor Vergata, University of Rome "Tor Vergata" – viale Oxford 81, 00133, Rome, Italy

^2^IRCCS Neuromed – via Atinense 18, 86077, Pozzilli (IS), Italy

^3^Department of Clinical Biochemistry and Molecular Biology, Policlinico Tor Vergata, University of Rome "Tor Vergata" – viale Oxford 81, 00133, Rome, Italy

^4^Neurology Unit, Policlinico Tor Vergata, University of Rome "Tor Vergata" – viale Oxford 81, 00133, Rome, Italy

*The authors contributed equally to this work.

**Corresponding Author:**

Dr. Caterina Motta

Memory Clinic and Neurodegenerative Dementia Research Unit

Policlinico Tor Vergata, University of Rome “Tor Vergata”

Viale Oxford, 81, Rome, 00133, Italy

email address: caterina.motta@ptvonline.it

tel. 0039 06 20903137

**Supplementary material**


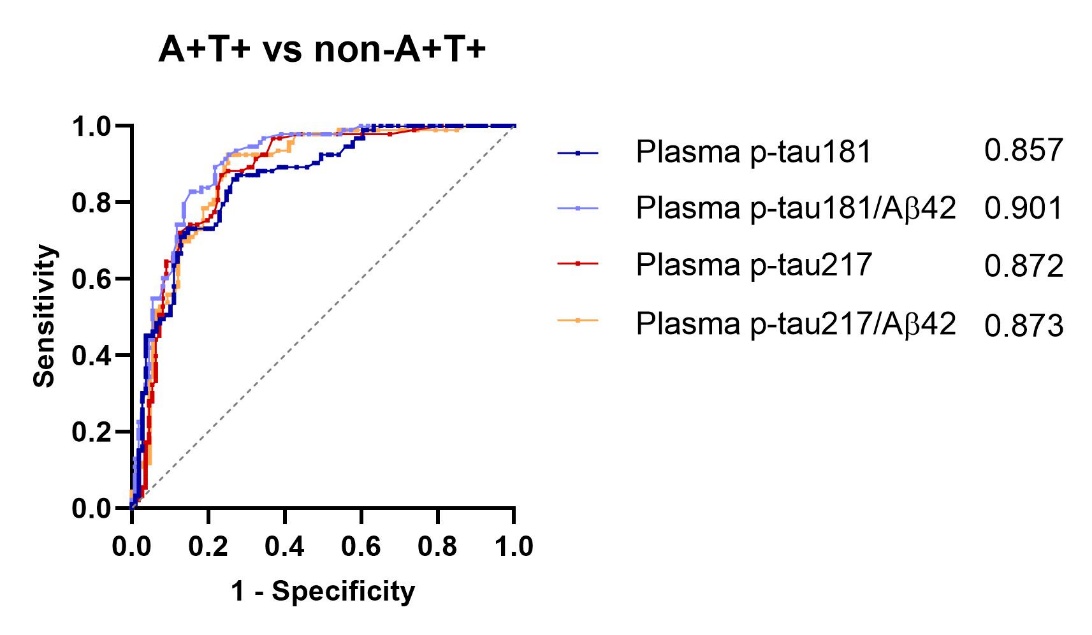


**Figure S1.** ROC curves for plasma p-tau181, plasma p-tau181/Aβ42, plasma p-tau217, and plasma p-tau217/Aβ42 in discriminating amyloid and tau positive (A+T+) versus non-A+T+ subjects. The corresponding AUC (area under the curve) values are shown to the right of each biomarker. p-tau = phosphorylated tau.
